# The Relationship between Body Mass Index, Obesity, and LINE-1 Methylation: A Cross-Sectional Study on Women from Southern Italy

**DOI:** 10.1155/2021/9910878

**Published:** 2021-12-03

**Authors:** Andrea Maugeri, Martina Barchitta, Roberta Magnano San Lio, Giuliana Favara, Claudia La Mastra, Maria Clara La Rosa, Antonella Agodi

**Affiliations:** Department of Medical and Surgical Sciences and Advanced Technologies “GF Ingrassia”, University of Catania, Catania 95123, Italy

## Abstract

Uncovering the relationship between body mass index (BMI) and DNA methylation could be useful to understand molecular mechanisms underpinning the effects of obesity. Here, we presented a cross-sectional study, aiming to evaluate the association of BMI and obesity with long interspersed nuclear elements (LINE-1) methylation, among 488 women from Catania, Italy. LINE-1 methylation was assessed in leukocyte DNA by pyrosequencing. We found a negative association between BMI and LINE-1 methylation level in both the unadjusted and adjusted linear regression models. Accordingly, obese women exhibited lower LINE-1 methylation level than their normal weight counterpart. This association was confirmed after adjusting for the effect of age, educational level, employment status, marital status, parity, menopause, and smoking status. Our findings were in line with previous evidence and encouraged further research to investigate the potential role of DNA methylation markers in the management of obesity.

## 1. Introduction

Overweight and obesity are delineated by an excessive accumulation of body fat, which results in a body mass index (BMI) greater than or equal to 25 kg/m^2^ and 30 kg/m^2^, respectively [1]. According to the most recent estimates by the World Health Organization (WHO), nearly 2 billion adults were overweight in 2016, out of which approximately 650 million were obese [2]. In line with these estimates, more than one adult in ten (~13%) were obese in 2016, with a prevalence that tripled in the last four decades [2]. The reasons behind this increment is probably attributable—at least in part—to the increased intake of energy-dense foods and to the increasingly sedentary nature of human life [2]. Overweight and obesity also account for an important burden for public health [3], because raised BMI is often associated with an increased risk of cardiovascular diseases, diabetes, musculoskeletal disorders, and some cancers [4]. It is also noteworthy that raised BMI could have adverse consequences on women of childbearing age and especially during pregnancy. For instance, excessive weight gain prior and during pregnancy was associated with adverse outcome in both mothers and their children [5–11]. Moreover, children born from overweight or obese women were not only at higher risk of being born large for gestational age [7, 9, 12, 13] and preterm [14] but also to develop metabolic disorders later in life [15–17].

In this complex scenario, it would be interesting to uncover molecular mechanisms associated with raised BMI and obesity. Among them, epigenetic mechanisms surely attracted the attention of many researchers, due to their potential role in development of obesity from the early stages of life [18]. For instance, previous studies already suggested the involvement of DNA methylation, aberrant miRNA expression, histone modification, and nucleosome release in obesity and associated comorbidities [19–21]. Specifically, DNA methylation is one of the best characterized epigenetic mechanisms, and previous studies investigated its association with cardiovascular diseases, obesity, diabetes, and cancer [22–24]. DNA methylation is generally regulated by different DNA methyltransferases (DNMTs), which are involved in several physiological and molecular processes, such as genomic imprinting, X-chromosome inactivation, gene expression, maintenance of chromosome integrity, and DNA-protein interactions [25]. Several studies used the methylation of long interspersed nuclear elements (LINE-1) sequences as a proxy of global DNA methylation level. Although there is still no consensus on the validity of measuring LINE-1 methylation as a surrogate marker, aberrant methylation of these sequences might influence both chromosomal stability and gene expression [26, 27]. Previous research suggested the potential relationship between obesity and LINE-1 methylation, but further investigation is still needed. Thus, the current cross-sectional study is aimed at assessing the association of BMI and obesity with LINE-1 methylation level among women from Catania, Italy.

## 2. Materials and Methods

### 2.1. Study Design

The population of the present cross-sectional study consisted of women from 15 to 85 years, who underwent routine physical examination at three clinical laboratories in Catania (Italy) from 2010 to 2017. Overall, the study was conducted on nonpregnant women without previous or current diagnosis of cancer, diabetes, cardiovascular, neurodegenerative, and autoimmune diseases. The study protocol was in accordance with the Declaration of Helsinki and approved by the ethics committees “Catania” and “Catania 2” with the following protocol numbers: 52/2010/VE, 16/2015/CECT2, and 227/2011/BE. All women who met inclusion criteria were invited to participate, after being informed of all aspects of the research protocol. Those who agreed to participate in the study had to sign a written informed consent. At recruitment, height and weight were measured to the nearest 1 cm and 1 kg, respectively, using a medical digital scale with meter. BMI was calculated as the ratio between weight (kg) and squared height (m^2^), and participants were categorized into underweight, normal weight, overweight, and obesity according to the WHO criteria [28]. At the same time, women provided a blood sample for DNA extraction and LINE-1 methylation assessment. Women with incomplete information on anthropometric measures and those who did not provide a blood sample were excluded from the current analysis.

### 2.2. LINE-1 Methylation Analysis

DNA extraction and the assessment of LINE-1 methylation were performed using standardized protocols [29]. In brief, DNA was extracted from leukocytes using the QIAamp DNA Mini Kit (Qiagen, Milan, Italy). Next, bisulphite conversion of 40 ng of the extracted DNA was performed using the EpiTect Bisulfite Kit (Qiagen, Milan, Italy). Specifically, the assessment of methylation levels was performed on three CpG sites within the LINE-1 sequence (GenBank Accession No. X58075). To do that, the LINE-1 sequence was amplified by Hot start PCR on the Eppendorf Mastercycler (Eppendorf, Milan, Italy). The PCR reaction was conducted in a final volume of 25 *μ*l, containing 1.5 *μ*l of bisulfite-converted DNA, 12.5 *μ*l of PyroMark PCR Master Mix 2x, 2.5 *μ*l of Coral Load Concentrate 10x, and 2 *μ*l of primers (0.2 *μ*M for each). The sequences of forward and reverse-biotinylated primer were 5′-TTTTGAGTTAGGTGTGGGATATA-3′ and 5′-biotin AAAATCAAAAAATTCC CTTTC-3′, respectively [29]. The PCR conditions were the following: 1 cycle at 95°C for 15 min, 40 cycles at 94°C for 30 s, 50°C for 30 s, 72°C for 30 s, and a final extension at 72°C for 10 min. Finally, the PCR products were sequenced by pyrosequencing on the PyroMark Q24 instrument (Qiagen, Milan, Italy), using 0.3 mM of the sequencing primer 5′-AGTTAGGTGTGGGATATAGT-3′. For each CpG site, methylation level was calculated as the percentage of methylated cytosines over all cytosines. All the protocols were performed according to the manufacturers' instructions, and each sample was analysed in triplicate. All the assays included a positive (100% methylated DNA) and a negative (0% methylated DNA) control, while failed assays were repeated. Intraobserver coefficient of variability between replicates was 2.2% (SD = 1.0%), as previously reported. For each sample, LINE-1 methylation level was calculated as the mean of methylation level of the three CpG sites [30].

### 2.3. Covariates

At the recruitment, information on socio-demographic and behavioral factors were collected through the administration of structured questionnaires. Specifically, educational level was classified as low (primary school diploma or none), medium (secondary school diploma), or high (bachelor's degree or higher). Women were also classified, according to their employment status, as employed (including both part-time and full-time employment) or unemployed (including housewives and retired). For each woman, we also collected information about family structure and specifically asking if women lived alone or in couple and if they had at least a child. Regarding smoking status, women were classified as current, former, and never smokers. Instead, dietary data were obtained using a semiquantitative Food Frequency Questionnaires (FFQ), from which we estimated total daily energy intake [31].

### 2.4. Statistical Analysis

Statistical analyses were performed on the STATA software (version SE 16.0, StataCorp, College Station, USA). Prior to analysis, quantitative variables were tested for normality using the Kolmogorov-Smirnov test. Descriptive statistics were used to summarize categorical variables (using frequency and percentage) and quantitative variables (using median and interquartile range (IQR)). All variables were compared across BMI categories using the Chi-squared test for categorical variables and the Kruskal–Wallis test for quantitative variables. The association of BMI with LINE-1 methylation was examined by simple linear regression and further adjusting for age, educational level, employment status, marital status, parity, menopause, and smoking status. Similarly, the association of BMI categories with LINE-1 methylation was examined using normal weight as the reference group in unadjusted and adjusted linear regression models. Results were reported as *β* coefficients and their standard error (SE). All the analyses were two-sided and performed with a significance level of 0.05.

## 3. Results

### 3.1. Population Characteristics

**Figure 1:**
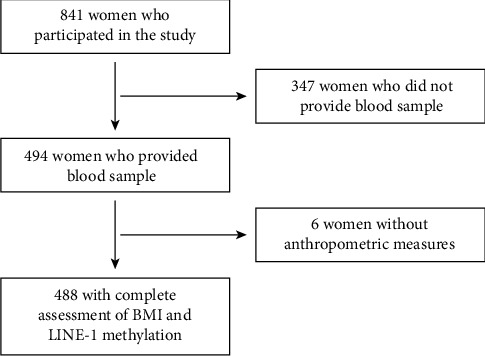
Flow chart of population selection.

Figure 1 describes the selection of participants according to inclusion and exclusion criteria. In brief, 494 out of 844 participating women provided a blood sample for the assessment of LINE-1 methylation. Among them, 6 women were excluded because of incomplete information on anthropometric measures. Thus, the study population consisted of 488 women, aged 15-85 years, with a complete assessment of anthropometric measures and LINE-1 methylation of leukocyte DNA. No differences between included and excluded women were evident. Regarding their education, 35.2% had a primary school diploma, 47.1% obtained a secondary school diploma, and 17.6% earned a degree. Overall, 44.1% of women were part-time or full-time employed, while 50.4% lived in couple. Approximately 70% had at least a child, while only 9.3% were menopausal. With respect to smoking status, most women never smoked (57.3%), a low proportion of them were former smokers (11.7%), and about one-third were current smokers (31.0%). The median total energy intake was 1935 kcal, and 17.4% used dietary supplements.

### 3.2. Comparisons across BMI Categories

According to their BMI (median of 23.3 kg/m^2^), women were classified as underweight (6.4%), normal weight (57.6%), overweight (23.6%), or obese (12.5%).  Table 1 compares the abovementioned characteristics across these BMI categories. Interestingly, the median age and hence also the proportion of menopausal women increased from the underweight to the obese category (*p* < 0.001 and *p* = 0.023). In line with increasing age, also the proportion of women who lived in couple and those who had at least a child increased (*p* < 0.001 and *p* = 0.004). Regarding social factors, the proportion of women with low educational level and those who were unemployed increased from the underweight to the obese category (*p* values < 0.001). With respect to behavioral information, the proportion of current smokers decreased from the underweight to the obese category (*p* < 0.001), while no differences were evident for total energy intake and use of dietary supplements.

### 3.3. The Relationship between BMI and LINE-1 Methylation

**Figure 2:**
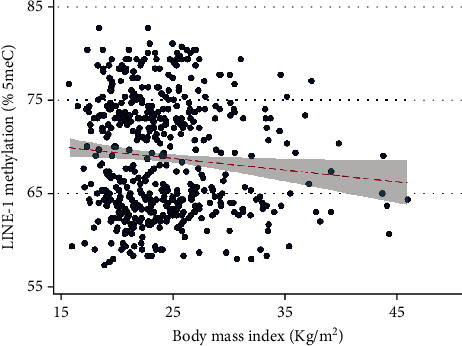
Scatter plot of the relationship between body mass index and LINE-1 methylation. The red line represents the linear regression line with its 95% confidence interval.

**Figure 3:**
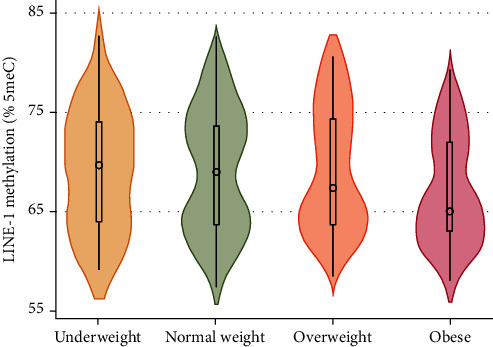
Violin plot showing the distribution of LINE-1 methylation level across categories of body mass index.

We first tested the relationship between BMI and LINE-1 methylation. As showed in the scatter plot reported in Figure 2, we noted a negative association so that the percentage of LINE-1 methylation decreased by 0.125 for each unit increase of BMI (SE = 0.057; *p* = 0.029). Accordingly, as depicted in the violin plot reported in Figure 3, we observed that LINE-1 methylation tended to decrease from the underweight to the obese category (*p* = 0.048). Indeed, median LINE-1 methylation level was 69.7 (IQR = 10.0) in underweight, 68.7 (10.0) in normal weight, 67.3 (10.7) in overweight, and 65.0 (IQR = 9.5) in obese women.

### 3.4. The Association of Obesity with LINE-1 Methylation

Finally, we tested the association of BMI and its categories with LINE-1 methylation level. To do that, we first adjusted the negative relationship between BMI and LINE-1 methylation for the potential effect of covariates (Table 2). Notably, the percentage of LINE-1 methylation significantly decreased by 0.145 for each unit increase of BMI (SE = 0.058; *p* = 0.013). Moreover, we evaluated the association between specific BMI categories and LINE-1 methylation, using normal weight women as the reference group. In the unadjusted model, obese women exhibited lower LINE-1 methylation level than their normal weight counterpart (*β* = −1.971; SE = 0.876; *p* = 0.025), while no significant differences were evident for underweight or overweight women. Interestingly, the negative association between obesity and LINE-1 methylation remained significant (*β* = −2.050; SE = 0.868; *p* = 0.019) after adjusting for age, educational level, employment and marital status, parity, menopause, and smoking habits.

## 4. Discussion

Our study demonstrated a negative relationship between BMI and LINE-1 methylation, which resulted in lower methylation level among obese women if compared with their normal weight counterpart. These findings were partially in line with the evidence summarized by a comprehensive review published by Samblas and colleagues in 2019 [18]. Indeed, several investigations have already suggested the relationship of weight gain and obesity traits with DNA methylation. Yet, these studies were heterogeneous in terms of study design, DNA source, methylation marker under investigation, and outcome of interest [18]. This produced a lot of findings, which, however, were not easy to interpret because in many cases they were often inconclusive or controversial. Indeed, obesity was associated with DNA methylation both positively and negatively, depending on the genes or DNA sequences under study [18].

To the best of our knowledge, few studies investigated the association between obesity and LINE-1 methylation. Among them, the cross-sectional study by Carraro and colleagues showed a positive association of waist circumference and BMI with methylation level in blood samples from 40 health professionals aged 20-59 years [32]. By contrast, a longitudinal analysis of the Bogota School Children Cohort demonstrated a negative association between adiposity measures and LINE-1 methylation in blood samples from children aged 5–12 years [33]. A negative association was also observed in our study, which for the first time evaluated the relationship between BMI, obesity, and LINE-1 methylation in a large population of women without previous or current diagnosis of severe diseases.

These controversies might be at least partially explained by the fact that several demographic, behavioral, and physiological factors could affect DNA methylation [18]. In the context of LINE-1 sequences, for example, it has already been demonstrated how nutrients, foods, and dietary patterns might influence methylation level. Specifically, the intake of nutrients involved in one-carbon metabolism, the consumption of fruits and vegetables, and the adherence to healthy dietary patterns appeared to be associated with higher LINE-1 methylation level [29]. In line with these findings, there was also evidence that weight loss interventions might significantly increase LINE-1 methylation level in blood samples [34, 35]. Moreover, it has been proposed that LINE-1 methylation level prior to the intervention might significantly predict the amount of weight loss [35]. Despite these interesting suggestions, however, there were also studies that produced inconclusive or opposite results [36, 37]. This was the case of the study by Martin-Nunez and colleagues, which instead showed a reduction in LINE-1 methylation level after an intervention promoting the adherence to the Mediterranean diet [36].

Although our findings confirm the relationship between obesity and LINE-1 methylation, their clinical utility for predicting obesity risk and response to weight loss programs is not immediate. In fact, the observational and cross-sectional nature of our study did not allow to establish a cause-effect relation. Specifically, further prospective studies should be encouraged to assess if LINE-1 methylation is a molecular mechanism underpinning obesity development or a consequence of this condition. Moreover, additional experimental research is necessary to confirm if LINE-1 methylation could be useful to predict weight loss in obese patients.

From a physiological point of view, several obesity-related factors were associated with aberrant DNA methylation. However, most of them were investigated only in vitro since they were challenging to be isolated in humans. Indeed, obese subjects often exhibited nutritional and physiological factors simultaneously, masking and/or confounding their independent effect on DNA methylation [18]. Despite these difficulties, it appeared clear that chronic inflammation [38, 39], oxidative stress [40], and insulin resistance [41] might play a key role in DNA methylation changes associated with obesity.

Our study had some limitations that should be considered when interpreting our findings. Firstly, the cross-sectional nature of our analysis did not allow to understand the causal relationship between obesity and LINE-1 methylation. Secondly, we used information on BMI and its classification, even if other anthropometric measures and adiposity indexes should have been considered additionally. For example, further studies should evaluate the association of LINE-1 methylation with several measures of fat deposition and abdominal obesity commonly used in epidemiological research [1, 18]. Thirdly, although our analyses were adjusted for several variables, other factors that could potentially affect DNA methylation and obesity (e.g., diet and physical activity) [42] should have been considered.

## 5. Conclusions

Our study demonstrated how increased BMI was associated with lower LINE-1 methylation level, especially in obese women. These findings—adding to the current knowledge on the relationship between obesity and DNA methylation—sustained the hypothesis that measuring obesity-related DNA methylation markers could be helpful to understand the molecular effects of inadequate weight gain. Moreover, it could be also useful for identifying people at higher risk of obesity or those who respond well to weight loss programs. However, at present, these are just interesting perspectives that merit further investigation through longitudinal and well-structured studies.

## Figures and Tables

**Table 1:** Characteristics of the study population across categories of the body mass index.

Characteristics	Underweight (*n* = 31)	Normal weight (*n* = 281)	Overweight (*n* = 115)	Obese (*n* = 61)	*p* value
Age, years	30 (11)	39 (18)	46 (22)	44 (21)	<0.001
Educational level					
Low	19.3%	30.4%	46.2%	53.9%	<0.001
Medium	49.1%	46.9%	44.0%	34.8%	
High	31.6%	22.7%	9.9%	11.2%	
Unemployed	45.6%	51.3%	59.9%	74.2%	<0.001
Living in couple	18.6%	46.1%	68.3%	75.6%	<0.001
Having children	36.8%	70.5%	80.9%	78.9%	0.004
Menopause	0.0%	15.2%	21.8%	15.8%	0.023
Smoking status					
Never smokers	47.7%	54.0%	60.4%	61.8%	<0.001
Former smokers	7.0%	7.9%	15.9%	13.5%	
Current smokers	45.6%	38.1%	23.6%	24.7%	
Total energy intake, kcal	2014 (705)	1923 (650)	1935 (708)	1950 (778)	0.335
Users of supplements	11.6%	15.7%	16.3%	15.6%	0.905

Results are reported as median (IQR) or percentage (%) and compared using the Kruskal-Wallis or the Chi-squared tests.

**Table 2:** Linear regression analyses between BMI, its categories, and LINE-1 methylation level.

Model^a^	BMI	*β* coefficient	Standard error	*p* value
Unadjusted	Continuous	-0.125	0.057	0.029
	Categories			
	Underweight	0.194	1.173	0.868
	Normal weight	Ref.		
	Overweight	0.170	0.687	0.803
	Obese	-1.971	0.876	0.025

Adjusted	Continuous	-0.145	0.058	0.013
	Categories			
	Underweight	-0.015	1.161	0.990
	Normal weight	Ref.		
	Overweight	-0.108	0.687	0.875
	Obese	-2.050	0.868	0.019

Results are reported as *β* coefficients, standard errors, and *p* values obtained through the linear regression analyses. The normal weight category was used as the reference group (Ref.) where indicated. The adjusted models included age, educational level, employment status, marital status, parity, menopause, and smoking status.

## Data Availability

The data used to support the findings of this study are available from the corresponding author upon request.
